# Selective Killing of Cancer Cells by Ashwagandha Leaf Extract and Its Component Withanone Involves ROS Signaling

**DOI:** 10.1371/journal.pone.0013536

**Published:** 2010-10-21

**Authors:** Nashi Widodo, Didik Priyandoko, Navjot Shah, Renu Wadhwa, Sunil C. Kaul

**Affiliations:** 1 National Institute of Advanced Industrial Science and Technology (AIST), Tsukuba, Ibaraki, Japan; 2 Department of Biology, Faculty of Mathematics and Natural Sciences, Brawijaya University, Malang, Indonesia; 3 Graduate School of Life and Environmental Sciences, University of Tsukuba, Tsukuba, Ibaraki, Japan; Indiana University, United States of America

## Abstract

**Background and Purpose:**

Ashwagandha is a popular Ayurvedic herb used in Indian traditional home medicine. It has been assigned a variety of health-promoting effects of which the mechanisms remain unknown. We previously reported the selective killing of cancer cells by leaf extract of Ashwagandha (i-Extract) and its purified component Withanone. In the present study, we investigated its mechanism by loss-of-function screening (abrogation of i-Extract induced cancer cell killing) of the cellular targets and gene pathways.

**Methodology/Principal Findings:**

Randomized ribozyme library was introduced into cancer cells prior to the treatment with i-Extract. Ribozymes were recovered from cells that survived the i-Extract treatment. Gene targets of the selected ribozymes (as predicted by database search) were analyzed by bioinformatics and pathway analyses. The targets were validated for their role in i-Extract induced selective killing of cancer cells by biochemical and molecular assays. Fifteen gene-targets were identified and were investigated for their role in specific cancer cell killing activity of i-Extract and its two major components (Withaferin A and Withanone) by undertaking the shRNA-mediated gene silencing approach. Bioinformatics on the selected gene-targets revealed the involvement of p53, apoptosis and insulin/IGF signaling pathways linked to the ROS signaling. We examined the involvement of ROS-signaling components (ROS levels, DNA damage, mitochondrial structure and membrane potential) and demonstrate that the selective killing of cancer cells is mediated by induction of oxidative stress.

**Conclusion:**

Ashwagandha leaf extract and Withanone cause selective killing of cancer cells by induction of ROS-signaling and hence are potential reagents that could be recruited for ROS-mediated cancer chemotherapy.

## Introduction

Indian traditional home medicine system, Ayurveda is renown for its oldest history in the world. Ashwagandha (*Withania somnifera*; Solanaceae) herb, proudly called Queen of Ayurveda, is one of the most commonly used plants in Ayurveda for a variety of effects that range from analgesic, astringent, adaptogen, anti-inflammatory, anti-stress, antispasmodic, anti-diabetes, immuno-stimulant and cardioprotective [1]–[4]. Extracts from different parts of the plant including root, shoot, seed and berry have been in use in various home-remedy recipes; root being particularly a common source for health-promoting alkaloids, withanolides and antioxidants. The main active constituents of Ashwagandha leaves are alkaloids and steroidal lactones (commonly known as Withanolides) [5]–[7]. We had previously investigated the biological activity in an alcoholic extract of Ashwagandha leaf extract (i-Extract) and demonstrated that it has a strong anti-cancer activity. By chemical fractionation, the activity was assigned to its constituent Withanone (i-Factor) that was shown to cause activation of tumor suppressor protein p53 in cancer cells only [8], [9]. Normal human fibroblasts showed down-regulation of p53 function and delayed senescence [10].

In the present study, we anticipated that the selective cancer cell-killing effect of i-Extract or Withanone is likely to be mediated by more than one genes/pathways and hence undertook an unbiased loss-of-function approach wherein gene knockdown was achieved by hammerhead ribozymes. Ribozyme population rescued from randomized ribozyme library-infected human breast cancer cells that survived the i-Extract treatment was characterized. Bioinformatics, biochemical and visual assays were employed to investigate the identified gene targets and to reveal their involvement in i-Extract-induced cancer cell killing. We demonstrate that the selective killing of cancer cells by i-Extract and its component Withanone involves ROS signaling.

## Results and Discussion

**Figure 1:**
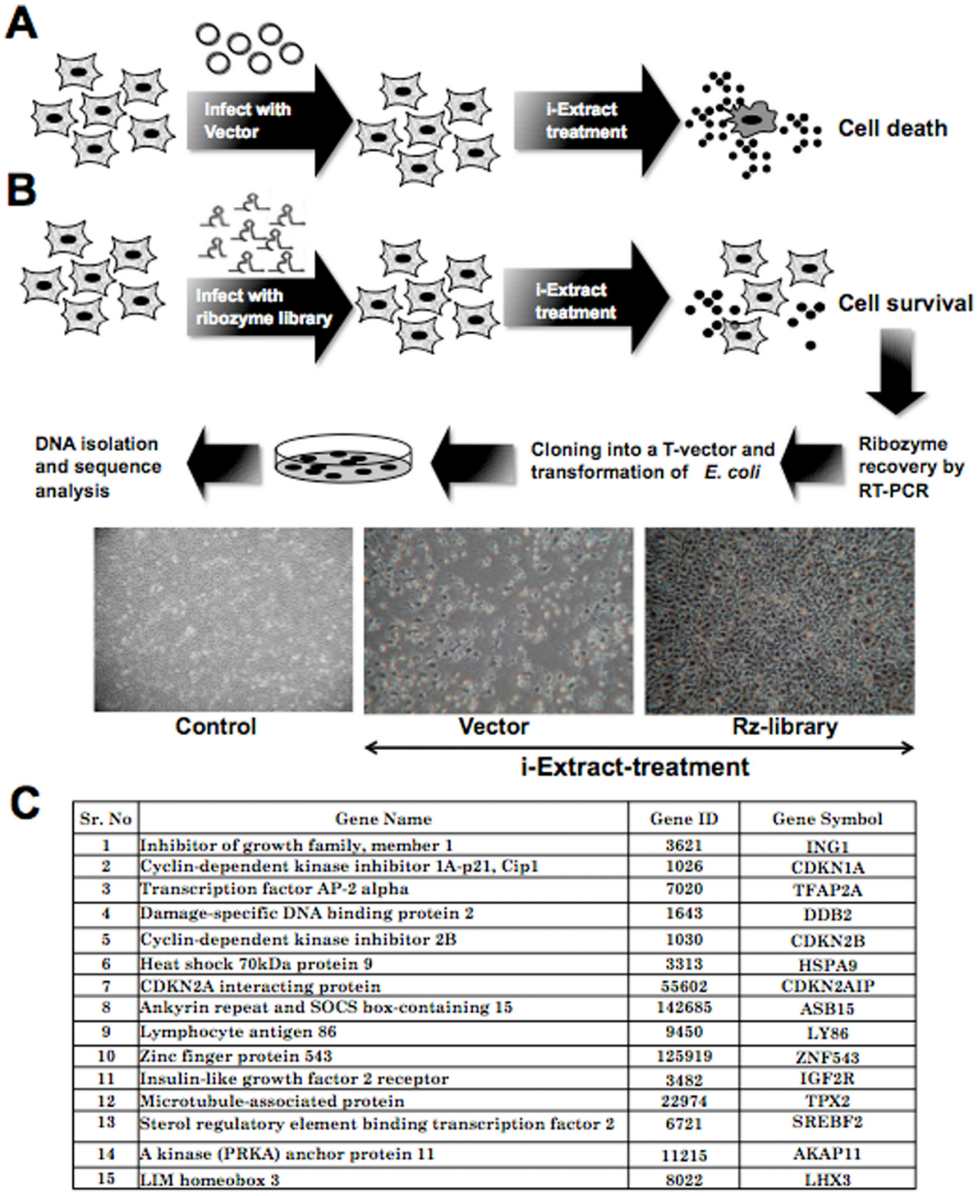
Screening for gene targets involved in i-Extract induced cyto-toxicity. Schematic presentation of loss-of-function screening using randomized ribozyme library. Control cells treated with i-Extract showed cytotoxicity (*A*). i-Extract treated surviving cells were collected (*B*). Ribozymes were rescued from the surviving cells by cloning and were characterized by sequence analysis (*B*). Candidate gene targets are shown in (*C*).

**Figure 2:**
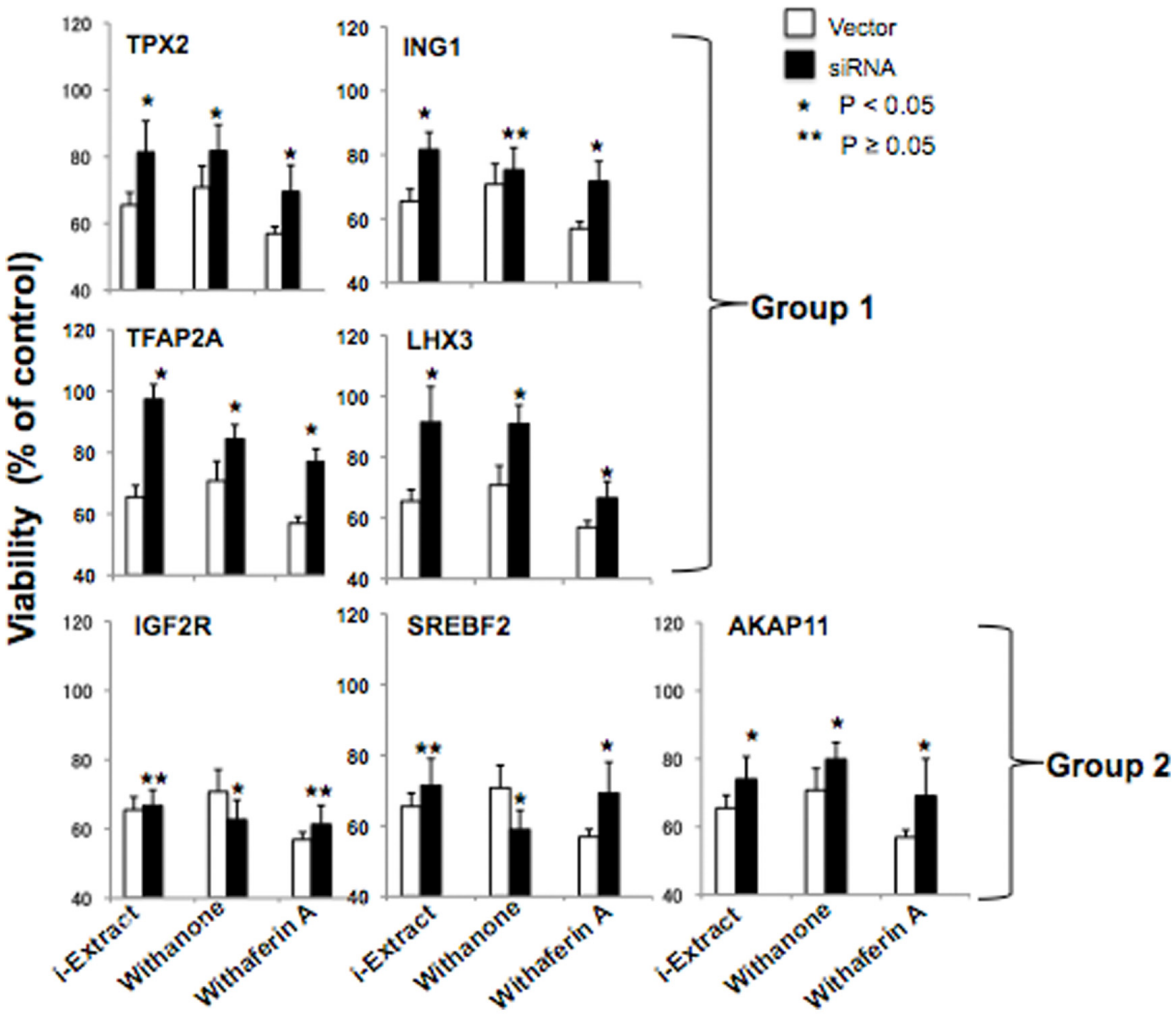
Effect of shRNA-mediated gene silencing on i-Extract induced cytotoxicity. Cells were transfected with vectors expressing shRNA for indicated genes. The effect of i-Extract was evaluated by cell viability assay. Results represent the mean of three experiments. Statistical significance was calculated by Analysis Of Variance (ANOVA) test.

Hammerhead ribozymes (HH-Rz) are the small catalytic RNAs that possess conserved hammerhead like secondary structure. They fold into the active conformation by binding to metal ions and hydrolyze phosphodiester bonds of RNA strands at specific sites (NUX, where N can be any nucleotide and X can be A, C or U) and hence are known as “RNA scissors” [11], [12]. HH-Rz have been in use as gene- silencing tools due to their features such as, high substrate binding specificity, autocatalytic activity that does not require other enzymes, flexible structure allowing manipulations and lack of interferon response in mammalian cells [13], [14]. In order to generate a gene specific ribozyme, its recognition arms (7–9 nucleotides flanking the target site) are designed to include sequence complementary to the target mRNA. Randomization of these 7–9 nucleotides in each arm yields a large variety of ribozyme capable of targeting multiple mRNA substrates [11]. Such pool of degenerate ribozymes expressed from an exogenous promoter has been used as a tool for identification of genes involved in apoptosis, migration, invasion, differentiation and diseases [15]–[20]. In order to identify the cellular targets involved in cancer cell cytotoxity of Ashwagandha leaf extract (i-Extract), we infected the MCF7 cells with retrovirus driven randomized ribozyme library prior to the treatment. As shown in Figure 1, when vector infected (control) cells treated with i-Extract resulted in cell death, ribozyme library infected culture showed cell survival. Ribozymes were rescued from these surviving cell population and characterized by cloning and sequence analysis. Gene targets for the isolated ribozyme sequences were determined by database search (http://blast.ncbi.nlm.nih.gov/Blast.cgi). The potential gene targets of the ribozyme sequences that were isolated multiple times are listed in Figure 1C. In order to validate the involvement of these genes in i-Extract induced cancer cell killing, we undertook two-way analyses (i) gene specific shRNA-mediated silencing and (ii) bioinformatics and systems biology directed pathway investigation. We prepared 7 gene (IGF2R, SREBF2, AKAP11, TFAP2A, LHX3, TPX2 and ING1) specific shRNAs and investigated their involvement in i-Extract and its components (Withanone and Withaferin A) induced MCF7 cell killing. Even under the efficiency of transfection ranging from 40–60%, as determined by transfection of a GFP-expressing plasmid, silencing of four genes (Group 1 - TPX2, ING1, TFAP2A and LHX3) was associated with significant (20–40%) increase in cell survival subsequent to the i-Extract treatment (Figure 2). The other three genes (Group 2 - IGF2R, SREBF2 and AKAP11) showed only minor (2–8%) increase in survival. Furthermore, cells compromised for the expression of four Group 1 genes escaped the cytotoxic effect of both Withanone and Withaferin A. These data suggested that these four genes mediate the cytotoxicity of i-Extract. However, they may not be critically involved in selective toxicity of i-Extract and Withanone to cancer cells as described in our earlier studies demonstrating the involvement of p53 tumor suppressor pathway in this phenomenon [8]. These data indicated that the cancer cell killing by i-Extract was mediated, at least in part, by TPX2, ING1, TFAP2A and LHX3. TPX2 is a microtubule-associated protein that functions as an allosteric regulator of Aurora-A, an oncogene with essential role in centrosome maturation and chromosome segregation during mitosis requiring assembly and maintenance of a bipolar spindle [21]–[23]. It is overexpressed in multiple human cancers and has been proposed as an attractive anti-cancer target [24]. TFAP2A plays crucial role in tumor growth and progression by regulation of E-cadherin, MMP-2, c-kit, p21^WAF-1^, HER-2, BCL-2, insulin like growth factor receptor-1 and Smad signaling [25]. LHX3 is a homeodomain transcription factor and plays positive role in embryonic development, cell fate determination and oncogenesis [26], [27]. On the other hand, ING1, an ING family protein, is involved in human cellular senescence, tumor suppression and apoptosis [28], [29]. ING1 has been shown to modulate p53 activity and its downstream effectors, p21^WAF1^ and Bax by acetylation and stabilization [30]. Taken together, the data suggested that the cancer cell killing by i-Extract might involve repression of TPX2, TFAP2A and LHX3, and activation of ING1 functions; the mechanism and selectivity to cancer cells still remian unclear.

**Figure 3:**
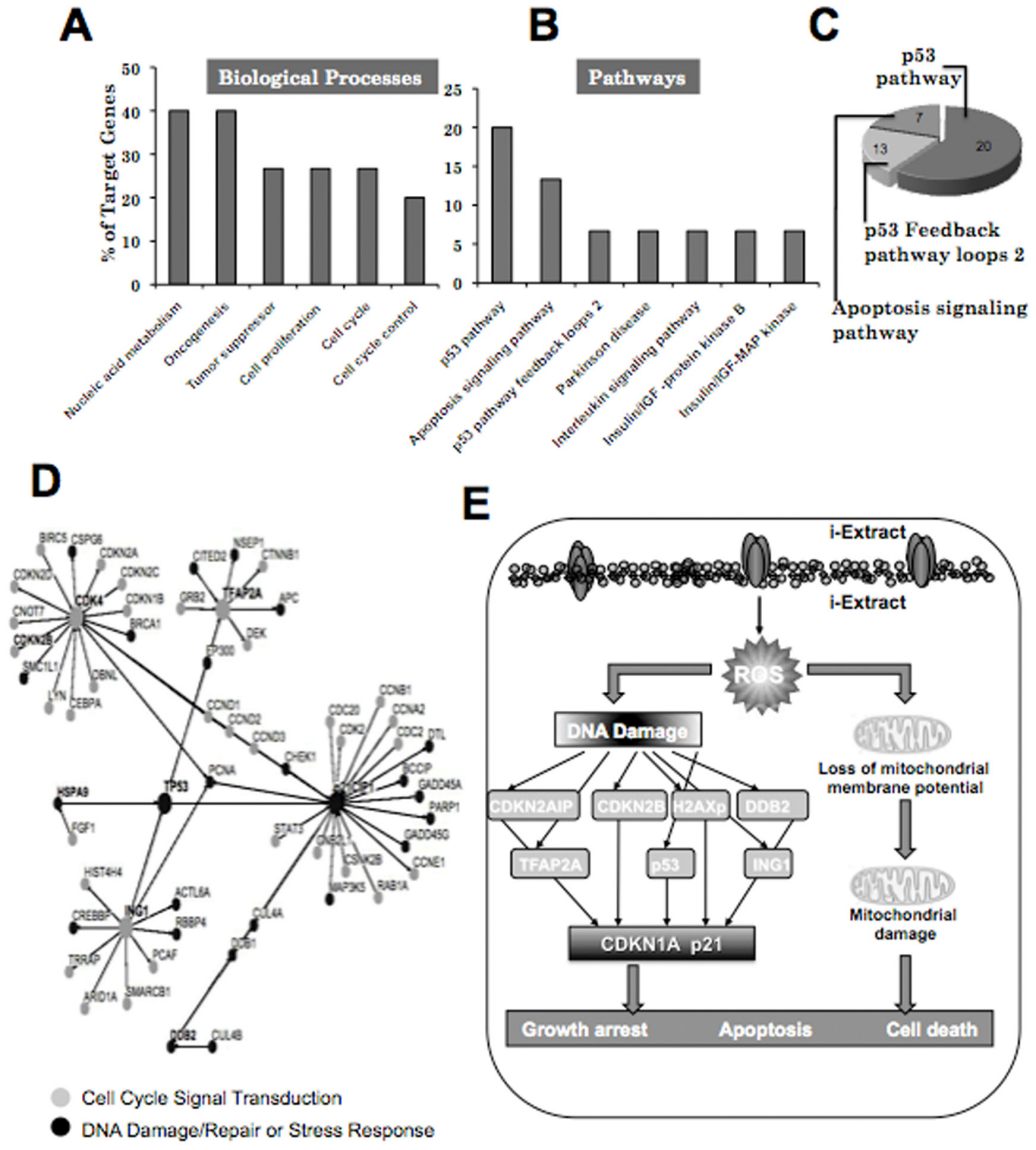
Bioinformatics analyses of the selected gene targets. Biological processes that were involved in i-Extract mediated cell death were predicted to include oncogenesis, cell proliferation and cell cycle (*A*) and involved p53, apoptosis, and insulin/IGF signaling pathways (*B*). Most predominant amongst these pathways were p53 and apoptosis (*C*). The target genes appeared as four clusters (CDK4, p21, ING1 and TFAP2A) that were connected by p53 and PCNA, involved in cell cycle, DNA damage response and DNA repair gene (*D*). Based on the selected gene targets, it was predicted that the i-Extract mediated cancer cell killing involved ROS- mediated damage at DNA and mitochondrial level with CDKN1A as a critical mediator (*E*).

**Figure 4:**
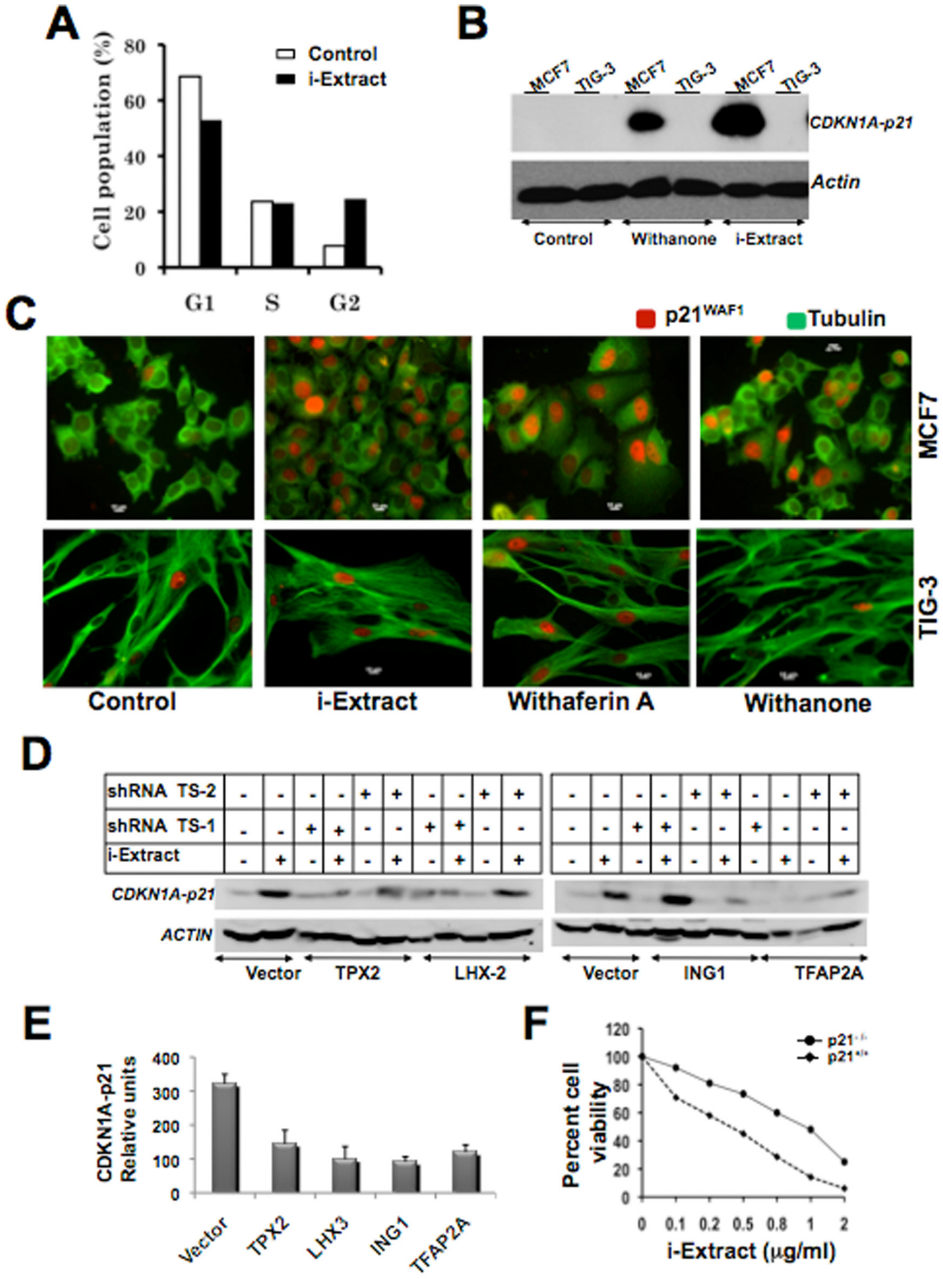
Role of CDKN1A-p21 in i-Extract induced cytotoxicity and its abrogation by selected gene targets. i-Extract induced growth arrest of MCF7 cells was due to their arrest at G2 cell cycle phase (*A*). An increase in CDKN1A-p21 expression was seen in MCF7 cells when treated with i-Extract and Withanone. No increase in CDKN1A-p21 expression was seen in normal cells in the presence of either i-Extract or Withanone (*B* and *C*). Knockdown of each of the four indicated genes that compromised i-Extract induced cytotoxicity also reverted the i-Extract induced CDKN1A-p21 upregulation (*D* and *E*). HCT116 p21^−/−^ cells were less sensitive to i-Extract as compared to their isogenic p21^+/+^ cells. Cell viability in response to the serially increasing concentrations of i-Extract (*F*).

In order to identify crucial cellular targets, we next undertook bioinformatics and systems biology approach and examined the network/pathways of the identified gene targets described above (Figure 3). The analyses revealed the involvement of isolated gene targets in several kinds of biological processes such as, oncogenesis, cell cycle, DNA repair and nucleic acid metabolism (Figure 3A). The top two identified pathways were p53 tumor suppressor (gene targets - DDB2, CDKN1A, CDKN2B) and apoptosis (gene targets - IGF2R and HSPA9). Of note, the analyses indicated that 33% of the genes involved in p53 pathway and its regulation, and 7% of the genes involved in apoptosis were identified suggesting that the cell killing by i-Extract involves growth arrest or apoptosis, mediated by activation of tumor suppressor p53 pathway. In addition, Ras, insulin/IGF, angiogenesis and cytoskeleton regulation pathways that are tightly linked with apoptosis and tumor development were also identified. These results demonstrated that the silencing of target genes abrogate the i-Extract mediated cell killing by protecting the cells from DNA damage, cell cycle arrest and apoptosis. Network interaction analysis of the target genes conceived four gene clusters - CDK4, TFAP2A, CDKN1A-p21 and ING1 linked by p53 and PCNA. Involvement of these gene clusters during i-Extract induced cytotoxicity suggests that it might be characterized as cellular responses, including stress response (HSPA9, CDKN1A) and DNA damage and repair response (ING1, DDB2 and TFAP2A), culminating into either cell cycle arrest or apoptosis (Figure 3D). Based on these parameters identified by bioinformatics analysis, we predicted that the i-Extract might cause an activation of cellular stress signaling by ROS-mediated pathways initiated at two levels (i) mitochondrial stress leading to change in membrane potential and (ii) DNA damage stress leading to activation of DNA damage and repair machinery (Figure 3E). Of note, most of the identified gene targets seemed to fit into the predicted signaling pathways (Figure 3E). In order to test this hypothesis, we investigated whether CDKNIA-p21 is the critical regulator of the i-Extract mediated cancer cell killing. As shown in Figure 4, i-Extract mediated growth arrest in MCF7 cells (Figure 4A) was associated with an activation of CDKN1A-p21 (Figure 4B). We also investigated to examine whether CDKN1A-p21 was a critical mediator of i-Extract induced selective cancer cell killing. As shown in Figures 4B and 4C, whereas CDKN1A-p21 was increased in MCF7 cells, it remained unaltered in normal (TIG-3) cells in response to either i-Extract or Withanone treatment. In contrast, Withaferin A caused cytotoxicity to both cancer and normal cell and was seen to activate CDKN1A-p21 (Figure 4C). We next investigated the activation of CDKN1A-p21 in control and i-Extract treated MCF7 cells following the transfections of four shRNAs that abrogated i-Extract induced MCF7 cell killing, at least in part (Figure 2). As shown in Figures 4D and 4E, we found that the i-Extract induced increase in CDKN1A-p21 expression was abrogated by knockdown of each of these four target genes. These data demonstrated that the CDKN1A-p21 is a critical mediator of i-Extract induced selective growth arrest in cancer cells. We finally tested this hypothesis by employing isogenic p53^+/+^, p53 ^−/−^, p21^+/+^ and p21^−/−^ HCT116 colon cancer cells. We found that whereas p53^+/+^ and p53^−/−^ cells showed comparable sensitivity to i-Extract (data not shown), the p21^−/−^ cells were two-three times more tolerant to i-Extract treatment as compared to the p21^+/+^ cells (Figure 4F) endorsing that CDKN1A-p21 is indeed a vital mediator of the growth arrest induced by i-Extract in cancer cells. The data was consistent with our model proposed in Figure 3E.

**Figure 5:**
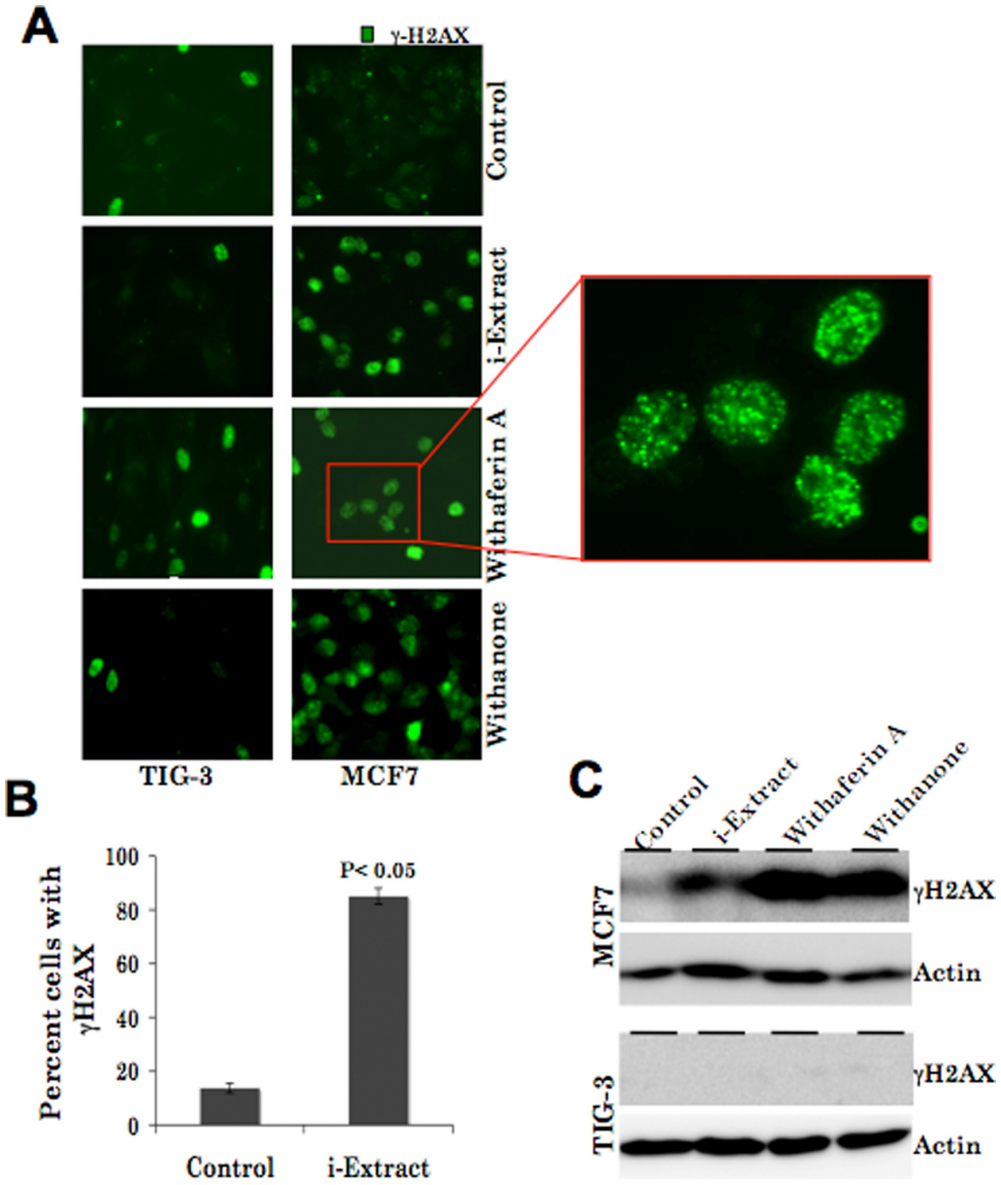
Role of DNA damage in i-Extract induced cytotoxicity. DNA damage foci (*A*), induction and γH2AX phosphorylation (*B*)-assessed by staining with phospho-specific antibody (100-200 cells per slide were counted) were detected in MCF7 cells treated with i-Extract, Withaferin A and Withanone. Normal cells showed DNA damage response upon the treatment with only Withaferin A (*A* and *C*).

As predicted by pathway analysis (Figure 3E), we next investigated the involvement of DNA damage and repair-signaling pathway in i-Extract induced cancer cell killing. As shown, MCF7 cells showed induction of γH2AX (an early marker of DNA damage response) in response to the treatment with i-Extract, Withaferin A and Withanone (Figures 5A and 5B). We also examined the phosphorylation of γH2AX by immunostaining with a phosphorylation specific antibody and found that the number of cells containing phosphorylated γH2AX was 70% higher in i-Extract treated cells as compared to the control (Figure 5B). Whereas the Withaferin A treatment induced γH2AX in both normal and cancer cells, i-Extract and Withanone induced γH2AX only in cancer cells (Figures 5A and 5C). Taken together, these data demonstrated that the selective cancer cell killing by i-Extract and Withanone was mediated by induction of DNA damage and CDKN1A-p21, as predicted in Figure 3E.

**Figure 6:**
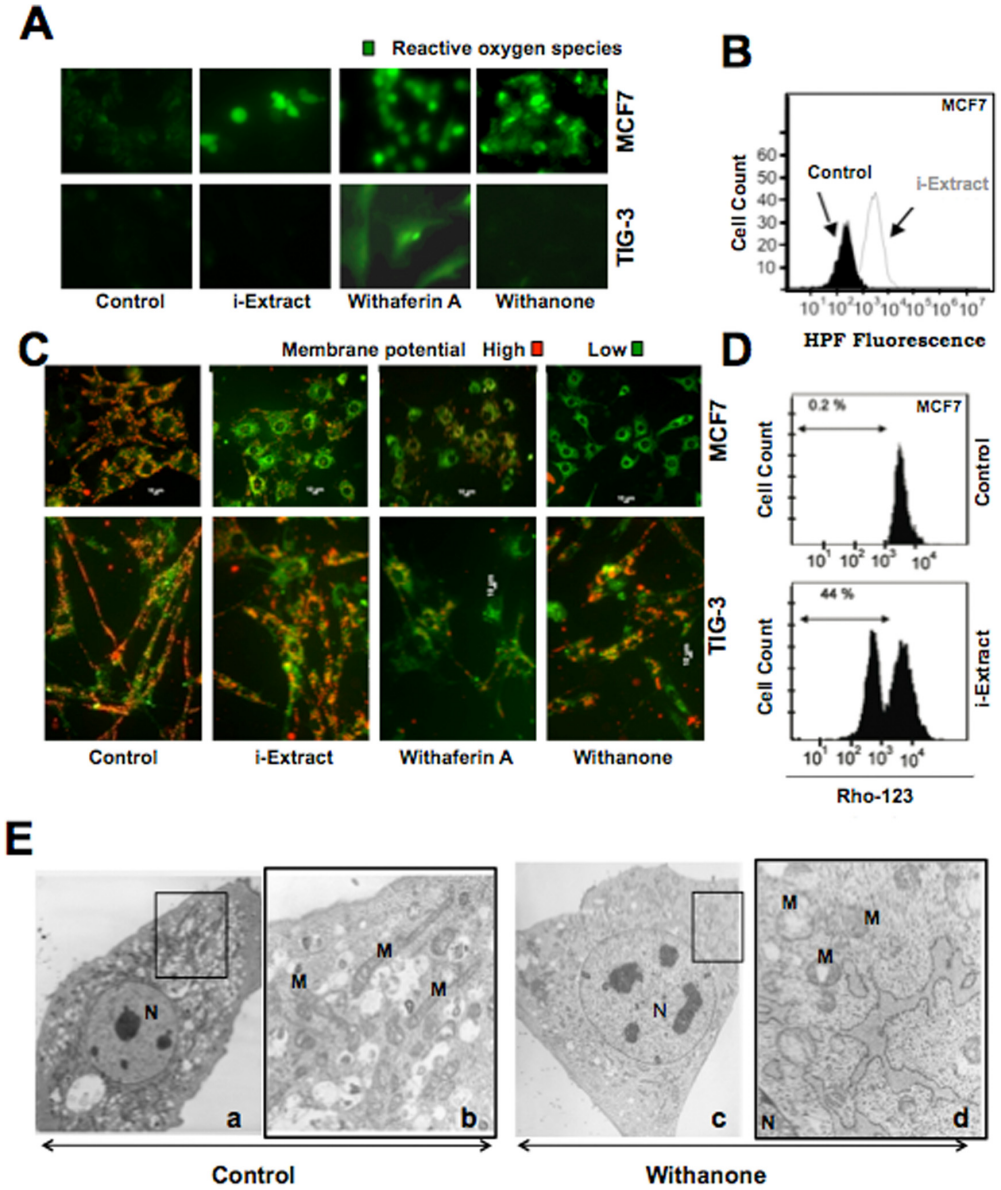
Role of ROS and mitochondrial damage in i-Extract induced cytotoxicity. MCF7 cells showed the induction of ROS when treated with i-Extract, Withaferin A or Withanone (*A* and *B*). Normal cells showed ROS induction only in the presence of Withaferin A (*A*). Loss of mitochondrial membrane potential in MCF7 cancer cells, as seen by JC-1 staining was detected with i-Extract only (*C*). Preferential induction of loss of mitochondrial trans-membrane potential in MCF7 cells detected by flow cytometry using RHO-123 increased from 0.2% of population in control to 44% of population treated with i-Extract (*D*). Mitochondrial damage was detected in Withanone-treated MCF7 cells. (*E*), Transmission electron microscopic images of control and Withanone-treated MCF7 cells. Control cells showed normal elongated mitochondrial (M) with parallel cristae (a) (enlarged boxed image, *b*), Withanone-treated cells showing swollen mitochondria with reduced number of the cristae (c) (enlarged boxed image, *d*). N, Nucleus.

We next investigated the involvement of stress response initiated at mitochondria in i-Extract induced MCF7 cell killing. As shown in Figures 6A and 6B, induction of ROS in the presence of i-Extract was detected, by both immunostaining and Fluorescence Probe (HPF) methods. Consistent with the cytotoxicity of the two components (Withaferin A and Withanone) of i-Extract, ROS induction was detected by both the components in MCF7 cells and only by Withaferin A in TIG-3 cells. These data demonstrated that the treatment with i-Extract and Withanone lead to induction of ROS, selectively in cancer cells, hence could be responsible for selective cancer cell killing. We next analyzed the mitochondrial membrane potential that is highly affected by ROS levels. As shown in Figures 6C and 6D, there was a decrease in mitochondrial membrane potential in MCF7 cells as seen by both JC-1 staining and Rho-123 exclusion assay. Upon treatment with i-Extract, MCF7 cells showed shift in JC-1 staining from red to green (Figure 6C) and negative for Rho-123 staining. Of note, normal (TIG-3) cells were refractory to these changes when treated with either i-Extract or Withanone (Figure 6C). These data made us to believe that the i-Extract induced selective killing of cancer cells involved ROS stress and mitochondrial damage. In order to obtain more clear evidence, we examined the ultrastructure of control and i-Extract treated MCF7 cells at single-cell level. As shown in Figure 6E, control MCF7 cells showed typical mitochondrial morphology, characterized by a double membrane containing a homogeneous matrix and a system of parallel cristae. Withanone treated cells showed swollen mitochondria with an altered morphology of which the shortening and reduction in number of cristae were apparent (Figure 6E).

ROS are chemically reactive molecules that have essential role in signal transduction, cell growth and differentiation, regulation of enzyme activities and immune response including inflammation and cytokine production. Whereas a moderate increase in ROS promotes cell proliferation and differentiation, its excessive amount causes irreversible oxidative damage to DNA, proteins and lipids leading to cell death. Cancer cells frequently exhibit high oxidative stress and increased generation of reactive oxygen species (ROS) as compared to the normal counterparts. This higher level of ROS serves as a selective therapeutic target by making cancer cells vulnerable to ROS-producing reagents, proposed as chemotherapeutic reagents [31]. A small number of studies have presented evidence to the ROS- mediated selective killing of cancer cells. These include, selective apoptosis in cancer cells by avocado extract [32], beta-phenylethyl isothiocyanate (PEITC) [33], thalidomide analogs [34] and MKT077 [35]–[36]. Interestingly, most of these reagents have been shown to cause mitochondrial disfunction [31]–[39]. We have demonstrated, for the first time, that the Ashwagandha leaf extract (i-Extract) and its component, Withanone act as ROS-producing agents causing DNA and mitochondrial damage discriminately to cancer cells, and hence are good candidates for safe cancer therapy.

## Materials and Methods

### Cells and randomized hammerhead ribozyme library screening

Human normal fibroblasts (TIG-3), breast carcinoma (MCF7), colon carcinoma (HCT116- p53^+/+^, p53^−/−^, p21^+/+^ and p21^−/−^) and mouse packaging cells, PLAT-E were obtained from Japanese Collection of Research Bioresources (JCRB) Cell Bank and cultured in Dulbecco's modified Eagle's minimal essential medium (DMEM) as described earlier [8]–[10]. Cells were treated with i-Extract, Withanone and Withaferin A for 48–72 h for viability, biochemical and visual analyses. Randomized ribozyme library (pMX-puro/Rz) was prepared and infected into MCF7 cells as described previously [15]. Infected cells were selected in puromycin (2 µg/ml)-supplemented medium for 24–48 h and were then treated with i-Extract. RNA was prepared, using Isogen (Invitrogen), from surviving cells after 4–5 days when all the cells in control dish had died. Total RNA (2 µg) was used for RT-PCR. Reverse transcription was performed with lower primer (5′-TTT TTT TTT TTT TTT TTT TTG GTA C-3′) and MMLV transcriptase (42°C, 90 mins) and the RT-product was subjected to PCR amplification using upper (5′-tcc ccg gtt cga aac cgg gca-3′) and lower primers (94°-52°-72°/30 sec each, 20 cycles). The amplified PCR product (∼150 bp) was cloned into a TA-cloning vector (Promega) and sequenced using T7 primer.

### Gene specific shRNA-vector cloning

shRNA expression vectors for 7 genes (IGF2R, SREBF2, AKAP11, TFAP2A, LHX3, TPX2 and ING1) were prepared as described [15]. Two target sites were selected for each of the gene. The target sequences selected for ING1 were ATATGAAGTTTAAATTCTA and GCCAAGACCTCCAAGAAGA, for TPX2 were GCAAGAAGGATGATATTAA and GGGGAAGAATGGAACTGGA, for TFAP2A were GGAGGAAGATCTTTAAGAG and GATCAAACTGTAATTAAGA, for AKAP11 were GAGTGAAGCTTTATCAAAT and GCACAAACATGGAAAGTCA, for SREBF2 were GCCTCAACCTCAAACTCAG and ATGCAAAGGTCAAAGATGA, for LHX3 were GCGACGAGTTCTACCTCAT and GGGAGAGCGTTTACTGCAA, and for IGF2R were GGCAGAAACCCAAACTGAA and GGAGGAAATACTACATTAA.

### Cell viability assay

Human breast cancer (MCF-7) cells were transfected with 50 ng of the indicated plasmid DNA. After 24 h, cells were treated with i-Extract (6 µg/ml), Withaferin A (1 µM), Withanone (25 µg/ml) for 48 h. Empty shRNA vector was used as a control. Viability of cells was monitored by WST-based cell proliferation assay (Roche). The doses for i-Extract and Withanone were selected based on the selective killing of cancer cells as described earlier (8–10). Lower doses of the i-Extract and Withanone were seen to induce differentiation of glioblastoma and neuroblastoma cells [40, and data not shown].

### Western blotting

The whole cell lysate (10–20 µg) in NP40 lysis buffer obtained by centrifugation at 10,000 rpm for 20 min at 4°C was used for immuno-blotting as described [8]. Antibodies used were p53 (DO-1; Santa Cruz Biotechnology), anti-mortalin [8], p21 (C-19; Santa Cruz Biotechnology) and anti-γH2AX-Phosphorylated (Ser139) antibody (Biolegend).

### Immunostaining

Cells were washed with phosphate buffered saline (PBS), fixed with methanol:acetone (1∶1), permeabilized in 0.2% PBST, blocked with 2% BSA (bovine serum albumin)/PBS and incubated with first and second antibody diluted in 2% BSA/PBS [8]. For γH2AX staining, the cells were fixed with 4% formaldehyde and permeabilized with KCM solution (120 mM KCl, 20 mM NaCl, 10 mM Tris pH 7.5, 0.1% Triton). Blocking and antibody preparation were done in ABDIL solution (2% BSA, 0.2% Gelatin, 150 mM NaCl, 0.1% Triton X-100, 20 mM Tris pH 7.5, 0.1% Sodium Azide in MilliQ water).

### Detection of reactive oxygen species

The reactive oxygen species were detected by fluorescent staining using the Image-iT™ LIVE Green Reactive Oxygen Species (ROS) Detection Kit (Molecular Probes Inc, USA). Cells were cultured on glass coverslips placed in 12-well plates and treated with indicated reagents for 48 h and stained for ROS following the procedure recommended by the manufacturers. Quantification of the ROS production with FACS Analysis was based on HPF (2-[6-(4′-hydroxy) phenoxy-3H-xanthen-3-0n-9-yl]benzoic acid) fluorescence. Briefly, MCF-7 cells were grown in 6-cm dish until 50% confluence, treated with i-Extract for 12 h followed by addition of HPF (10 mM). After 4 h, cells were washed with PBS, harvested with cell scraper. Cell fluorescence was measured by Coulter Epic XL Flow cytometer (Beckman).

### Mitochondrial membrane potential

Mitochondrial membrane potential for control and treated cells was examined by JC-1 and RHO-123 [35] based cell-staining assays. For JC-1 staining, MCF7 cells cultured in 24-well plates (50–60% confluency) were treated with i-Extract for 48 h followed by incubation with JC-1 stain (10 µg/ml) for 15 min at 37°C in CO_2_ incubator. The cells were washed with PBS and processed for microscopy. For RHO-123 assay, MCF-7 cell were grown in 6-cm dish until 50% confluence, treated with i-Extract for 48 h. The treated cells were incubated with Rho123 (1 mM)-supplemented medium for 1 h. Cells were washed with PBS and harvested with cell-scraper. Changes in mitochondrial membrane potential were monitored by Coulter Epic XL Flow cytometer (Beckman).

### Single cell ultrastructural analysis

MCF7 cells were plated on glass coverslips. At 60% confluency, cells were treated with Withanone for 24 h. Control and treated cells were washed with PBS and then fixed with 1.2% glutarldehyde in 4°C for 1 h. After post-fixation with 1% OsO_4_ at 4°C for 1 h, cells were washed in PBS and dehydrated through graded ethanol concentrations with final incubation in n-Butyl glycidyel ether for 15 min. Samples were embedded in Epoxy resin (TAAB) and were cut into ultra-thin sections with a Reichert ultramicrotome (Leica). The sections were stained with uranyl acetate and lead citrate and examined with a Hitachi H-7000 electron microscope. To evaluate mitochondrial alterations, ∼50 cells per sample were observed from two independent experiments.

### Bioinformatics and statistical analyses

Pathway analysis and biological process association were mapped using PANTHER-Expression data analysis [41], [42], a conceptually simple binomial test to compare classifications of multiple clusters of selected list with a reference list (NCBI: *Homo sapiens* Genes) to statistically determine over- or under- representation of PANTHER classification categories. Biological process result was selected based on over-representation value and P value less than 0.5. Protein-protein interactions for target genes were performed with Osprey based on the General Respository for Interaction Datasets (The GRID) and Gene onthology (GO)[43].
